# Indigenous Knowledge and Food‐Based Coping Mechanisms for ART Side Effects: A Qualitative Inquiry

**DOI:** 10.1155/arat/7724391

**Published:** 2026-10-04

**Authors:** Kasim Abdulai, Ivan Addae-Mensah, Angela Kyerewaa Aidoo, Mansura Ama Abakah, Adele Naa Ashikwei, Mary Foriwaa Fosu, Adjoa Antwiwaa Kye-Duodu

**Affiliations:** ^1^ Translational Nutrition Research Group, Department of Nutrition and Dietetics School of Allied Health Sciences University of Cape Coast Cape Coast Ghana ucc.edu.gh; ^2^ HopeSpring Harvest Foundation Cape Coast Ghana; ^3^ Department of Population and Health Faculty of Social Sciences University of Cape Coast Cape Coast Ghana ucc.edu.gh

**Keywords:** ART adherence, food insecurity, HIV, indigenous knowledge, qualitative research

## Abstract

**Introduction:**

Sustained adherence to antiretroviral therapy (ART) is critical for managing HIV, yet side effects and food insecurity present significant barriers in resource‐limited settings. While the role of formal support is recognized, the use of indigenous knowledge and food‐based coping mechanisms remains an underdocumented facet of self‐management. This study explored how individuals in Ghana utilize local foods and cultural knowledge to manage ART side effects and support adherence.

**Methods:**

A qualitative descriptive study was conducted at the Cape Coast Teaching Hospital. Through semistructured in‐depth interviews (*n* = 20) and one focus group discussion (*n* = 5) with adults on ART for at least 6 months, we collected rich, experiential data. Participants were purposively sampled, and data collection continued until thematic saturation was achieved. Thematic analysis was employed to identify patterns and themes related to food‐based coping strategies.

**Results:**

Analysis revealed that participants strategically employed indigenous knowledge, using specific local foods as active agents to manage side effects. Ginger tea was used to combat nausea, while avocado and groundnuts served as a protective stomach buffer. The timing of medication was often deliberately aligned with meals, with participants delaying doses if food was unavailable to avoid distress. These practices were deeply pragmatic, relying on affordable staples like gari (cassava flour) and yams, and were seamlessly integrated into daily routines. While these strategies demonstrated significant patient agency and resilience, they were also born from necessity, often representing suboptimal adaptations to food insecurity and poverty.

**Conclusion:**

Indigenous knowledge provides a crucial, culturally resonant toolkit for managing ART side effects and facilitating adherence. Healthcare systems should move beyond generic instructions to formally recognize, validate, and integrate these patient‐derived strategies into adherence counseling and peer support, fostering a more holistic and effective model of HIV care that bridges biomedical and indigenous knowledge systems.

## 1. Introduction

The global scale‐up of antiretroviral therapy (ART) has transformed HIV from a fatal diagnosis into a manageable chronic condition, enabling millions of people living with HIV (PLHIV) to lead long and healthy lives [1]. The clinical success of ART, however, is unequivocally contingent upon sustained and near‐perfect adherence, a benchmark that proves profoundly challenging for many individuals in resource‐limited settings [2]. While the barriers to adherence are multifaceted, encompassing structural, economic, and social factors, the management of drug‐related side effects remains a critical and often debilitating hurdle [3]. Symptoms such as nausea, dizziness, fatigue, and gastrointestinal distress are frequently reported, particularly during the initial phases of treatment or following regimen changes. For many, these adverse effects are not merely uncomfortable but are a primary reason for missed doses, treatment interruptions, and the subsequent risk of virologic failure and drug resistance, thereby undermining the very public health goals of treatment expansion.

The challenge of side‐effect management is acutely magnified within contexts of pervasive poverty and food insecurity. The pharmacological imperative to take ART with food to mitigate its side effects collides with the socioeconomic reality for many PLHIV, for whom a consistent and nutritious meal is not a guarantee. This intersection creates a cruel paradox: the medication essential for survival induces sensations that are intolerable without adequate nutrition, which the very condition and its treatment can impede one’s ability to secure. Consequently, individuals are forced into difficult risk–benefit calculations, often delaying or skipping doses to avoid the physical distress of taking powerful drugs on an empty stomach [4]. This decision is not born of negligence but is a rational, if perilous, coping strategy in the face of constrained choices, highlighting a critical gap in the standard biomedical model of care [5].

In response to this gap, a growing body of literature acknowledges the role of social support and economic interventions in facilitating adherence [6]. However, there remains a significant blind spot in mainstream HIV research regarding the agency and resourcefulness of PLHIV themselves, particularly their utilization of culturally embedded, indigenous knowledge systems [7]. Indigenous knowledge, understood as the cumulative, body of wisdom, practices, and beliefs evolved by communities through generations of interaction with their local environment, represents a vast, yet under‐documented, repository of strategies for health management. Within the realm of HIV care, this often manifests as the use of local foods and dietary adaptations to manage well‐being, a domain that has not been systematically explored as a formal adherence mechanism.

Indigenous knowledge, in the context of this study, refers to the cumulative body of strategies, practices, and beliefs about local food resources that have been developed by communities and transmitted across generations [8]. This knowledge is not merely cultural folklore but represents a practical, evidence‐based understanding of how specific foods, such as ginger for nausea or avocado for gastric protection, can be used to manage physical symptoms. Food‐based coping mechanisms, therefore, encompass the active, strategic use of these locally available foods to prevent, mitigate, or manage the side effects of biomedical treatments. A detailed operational definition of these concepts is provided in the Methods section, 2.6.

Across the globe, communities have long used food not only as sustenance but as medicine, possessing deep knowledge of the properties of local flora and foodstuffs to treat common ailments. This paradigm of “food as medicine” is highly relevant to the experience of PLHIV navigating ART side effects. Emerging evidence from sub‐Saharan Africa has documented the widespread use of traditional foods and herbal preparations among PLHIV to manage ART side effects [9]. Preliminary evidence and anecdotal reports suggest that individuals independently discover and share knowledge about specific foods, spices, and preparation methods that can soothe nausea, stabilize dizziness, or provide a protective buffer for the stomach. Whether it is the use of ginger for its antiemetic properties, the consumption of fatty seeds to slow gastric absorption, or the reliance on specific staple carbohydrates for their digestibility, these practices represent a sophisticated, patient‐driven form of symptom management. A study conducted in South Africa found that PLHIV frequently used traditional medicines and dietary modifications to manage ART side effects, with many reporting improved symptom control and treatment adherence [9]. Similarly, research in Ghana has documented that PLHIV practice food elimination, substitution, and supplementation to manage ART‐related symptoms, demonstrating a systematic approach to dietary self‐management [10]. They are a testament to the resilience and expertise developed at the grassroots level.

The concept of “food as medicine” is deeply rooted in many traditional healing systems and is increasingly recognized in biomedical literature. This paradigm posits that food possesses therapeutic properties beyond basic nutrition, capable of preventing, managing, or treating various health conditions [11]. In the context of HIV care, this concept is particularly relevant as PLHIV often experience malnutrition, weight loss, and gastrointestinal disturbances that can be mitigated through strategic dietary choices [12]. Traditional food systems, which incorporate locally available ingredients with known medicinal properties, offer a sustainable and culturally appropriate approach to managing treatment‐related side effects [10]. The integration of food‐based therapies with biomedical treatment represents a synergistic model that respects both cultural practices and clinical efficacy.

Therefore, this study seeks to illuminate these nuanced, bottom‐up coping strategies by investigating the indigenous knowledge and food‐based mechanisms employed by PLHIV to manage ART side effects. Moving beyond a deficit‐based narrative that frames patients solely as victims of structural constraints, we position them as active agents in their care, possessors of valuable knowledge that is pragmatic, accessible, and culturally resonant. Our research is guided by the central question: How do individuals living with HIV utilize indigenous knowledge and food‐based strategies to cope with ART side effects and support adherence in a resource‐limited setting? By answering this, we aim to bring to the forefront a critical dimension of lived experience that is often overlooked in clinical and policy dialogs.

To contextualize this inquiry, it is important to note that existing research has begun to document the role of indigenous food knowledge in HIV care. A study in KwaZulu‐Natal, South Africa, found that PLHIV commonly used traditional foods and herbal remedies alongside ART, with many reporting that these practices helped manage treatment side effects and improved their overall wellbeing [9, 13]. Similarly, research from Ghana has documented that PLHIV engage in food elimination, substitution, and nutrient supplementation practices specifically in response to ART side effects, indicating a systematic, patient‐driven approach to dietary self‐management [10]. In Uganda, traditional healers and community elders were identified as key sources of knowledge about indigenous foods with nutritional and medicinal properties for managing HIV‐related symptoms [14]. These studies suggest that indigenous knowledge systems represent a valuable, yet underutilized, resource for supporting ART adherence in resource‐limited settings.

Documenting these practices is not merely an academic exercise; it has profound implications for the development of more holistic, culturally competent, and effective HIV care models. Understanding these coping mechanisms can inform the counseling provided by healthcare workers, making it more practical and relatable. It can shape the composition of nutritional support packages and guide the discussions within peer support groups, fostering the sharing of effective, locally viable strategies [6]. Ultimately, by validating and integrating this indigenous knowledge into the formal healthcare system, we can work toward a more synergistic model of care, one that respects patient autonomy, leverages community assets, and bridges the critical gap between biomedical prescription and the daily realities of those it seeks to serve [7].

## 2. Methods and Materials

### 2.1. Study Setting

The study was conducted at the Cape Coast Teaching Hospital (CCTH) in the Central Region of Ghana. This region is characterized by a rich cultural heritage with strong ties to indigenous Akan (Fante) traditions, including a deep knowledge of local flora and their uses for food and wellness. CCTH is a major referral hospital providing comprehensive HIV care, including ART initiation and refills, counseling, and management of opportunistic infections, serving a large population of people living with HIV (PLHIV) from both urban and peri‐urban communities [15]. The setting was selected for its high patient load and the potential to access individuals with extensive local knowledge, which was crucial for investigating the study’s focus on indigenous food‐based coping mechanisms.

### 2.2. Research Design, Sampling, Sample, and Sample Size

This study utilized a qualitative descriptive research design, a methodology successfully employed in similar studies to gain an in‐depth understanding of complex health behaviors in Ghana [16]. Twenty‐one (21) adults living with HIV (aged 18 years and above) who had been on ART for at least 6 months and were long‐term residents knowledgeable about local traditions were purposively selected to participate. Participants’ duration on ART ranged from 6 months to over 10 years, with a mean duration of 4.2 years (SD = 3.1). This variation allowed for exploration of how indigenous knowledge accumulation and refinement occurred over the course of treatment. However, those who were critically sick had been on ART for less than 6 months, were recent migrants to the area, or could not communicate in English or Fante were excluded from the study (*n* = 02). To obtain rich, in‐depth data, the study employed a combination of twenty (20) in‐depth interviews (IDIs) and one (1) focus group discussion (FGD). The sample size was not predetermined but was guided by the principle of thematic saturation, which was determined to be achieved after the 20th interview and the subsequent FGD, as no new themes regarding food‐based coping mechanisms emerged. For sociodemographic characterization, data from 20 participants with complete information were used.

### 2.3. Data Collection Procedure

Data were collected over a period of 4 months in 2025. After ethical approval was obtained from the Cape Coast Teaching Hospital Institutional Review Board, potential participants were identified and referred by ART clinic staff. The research team then screened them against the inclusion criteria. Eligible individuals who expressed interest were provided with a detailed verbal and written explanation of the study using an information sheet. Those who agreed to participate provided written informed consent. In‐depth interviews and the focus group discussion were conducted in a private room at the clinic by trained qualitative researchers. A semi‐structured interview guide with open‐ended questions was used to explore participants’ experiences with ART side effects, the specific indigenous foods and preparations they used for management, the sources of this knowledge, and their perceptions of efficacy. The guide was developed based on study objectives, piloted for clarity, and translated between English and Fante as needed. Interviews and the FGD were audio‐recorded with permission, and each session lasted approximately 45–60 min. Field notes were taken to capture nonverbal cues and contextual observations.

To establish rapport and facilitate deep exploration of participants’ experiences, the research team employed several strategies. First, interviews were conducted in a private, comfortable setting at the clinic to ensure confidentiality and minimize distractions. Second, interviewers were trained in qualitative interviewing techniques, including active listening, open‐ended questioning, and the use of probes to encourage participants to elaborate on their responses. For example, when participants mentioned using a specific food, interviewers would probe with questions such as “Can you tell me more about how you prepare that?” or “How did you learn about that particular food?” Third, interviewers adopted a nonjudgmental and empathetic approach, validating participants’ experiences and knowledge to build trust. Participants were assured that there were no right or wrong answers and that their perspectives were valued. Field notes were used to capture nonverbal cues and contextual information that enriched the data.

The semi‐structured interview guide included the following main questions:

1. “Can you tell me about your experience with taking ART medication? What has it been like for you?”
2. “Have you experienced any side effects from your ART medication? If so, can you describe them?”
3. “What do you do to manage these side effects? Are there specific foods or drinks you use?”
4. “How did you learn about using these foods to manage your side effects? Who taught you?”
5. “How effective do you find these strategies? Do they help you take your medication consistently?”
6. “Are there times when you cannot use these strategies? What happens then?”
7. “What other sources of support do you have for managing your treatment?”

### 2.4. Data Analysis

Data were inductively analyzed using thematic analysis, following the six‐phase framework by Braun and Clarke. Audio recordings were transcribed verbatim and reviewed for accuracy. The analysis process involved repeated reading of transcripts to ensure familiarity with the data. Initial codes were generated manually and with the aid of NVivo software (version 12), focusing on specific foods, side effects, and knowledge transmission. These codes were then collated into potential themes, which were reviewed, refined, and defined through a collaborative process among the research team. A final thematic framework was developed, weaving the themes into a coherent narrative about indigenous coping strategies, supported by direct excerpts from the participants.

### 2.5. Ethical Consideration

We conducted this study under the principles of the Declaration of Helsinki. Ethical approval was obtained from the Cape Coast Teaching Hospital Institutional Review Board (CCTH/IRB/2023/012). Written informed consent was sought from all participants after a comprehensive explanation of the study. For illiterate participants, the consent form was read aloud in the presence of a witness, and a thumbprint was obtained. All interviews and discussions were conducted in a private room, and strict confidentiality was maintained through the use of anonymized participant ID codes. All electronic data were stored on password‐protected computers. Participants were informed that they could withdraw at any time without any consequence to their medical care. To protect cultural heritage, interviewers were trained to approach the topic of indigenous knowledge with sensitivity and respect.

### 2.6. Study Concepts

#### 2.6.1. Indigenous Knowledge

For this study, indigenous knowledge was defined as the cumulative body of strategies, practices, beliefs, and understandings about the use of local food resources developed by a community and passed down through generations. This knowledge is context‐specific and culturally embedded [8]. For this study, indigenous knowledge was operationalized as the practical, community‐derived understanding of local food properties and their application to health management. This included not only the identification of specific foods but also knowledge of preparation methods, optimal timing of consumption, and perceived efficacy, all of which are transmitted through family networks, community elders, and peer sharing. It was operationalized by asking participants to describe the specific local foods, preparation methods (e.g., teas, soups, specific combinations), and the rationale behind their use for managing ART side effects, as well as the sources from which they learned these practices (e.g., family elders, community members, peer patients).

#### 2.6.2. Food‐Based Coping Mechanisms

Food‐based coping mechanisms were defined as the active and strategic use of locally available foods, beverages, or food‐related practices to prevent, mitigate, or manage the physical side effects of ART, such as nausea, dizziness, stomach pain, and fatigue. This is a recognized challenge, as food insecurity has been shown to significantly hinder adherence to ART [17]. This was measured by prompting participants to describe their personal experiences: the specific side effects they encountered, the exact food or drink they consumed in response (e.g., ginger tea, avocado, gari), the timing of consumption in relation to medication, and their perceived effectiveness of these strategies in enabling them to maintain their ART adherence [10].

## 3. Results

### 3.1. Sociodemographic Characteristics of the Study Sample

The sociodemographic characteristics of the study participants (*N* = 20) are presented in Table 1. The sample was predominantly female and of working age, with generally low educational attainment and income levels highly variable but indicative of widespread financial precarity. This socioeconomic context is explored qualitatively in the first thematic finding below. The majority of participants (65%) had been on ART for more than 3 years, with 35% having received treatment for over 5 years. This long‐term exposure to ART provided participants with extensive experience in managing side effects and developing effective coping strategies over time.

**TABLE 1:** Sociodemographic characteristics of the study sample (*N* = 20).

Characteristic	Value
Age (years)	
Mean (SD)	41.90 (12.56)
Range	17–62
Gender, *n* (%)	
Female	17 (85.0)
Male	3 (15.0)
Duration on ART, *n* (%)	
Less than a year	2 (10.0)
1–3 years	5 (25.0)
3–5 years	6 (30.0)
More than 5 years	7 (35.0)
Marital status, *n* (%)	
Married	8 (40.0)
Single	6 (30.0)
Divorced	4 (20.0)
Widowed	2 (10.0)
Cohabiting	1 (5.0)
Highest education, *n* (%)	
Primary	6 (30.0)
Junior high school (JHS)	5 (25.0)
Senior high school (SHS)	3 (15.0)
Tertiary (Diploma/BSc)	3 (15.0)
Higher^a^	2 (10.0)
No formal schooling	1 (5.0)
Occupation, *n* (%)	
Trader	8 (40.0)
Unemployed	3 (15.0)
Civil servant	3 (15.0)
Farmer	3 (15.0)
Self‐employed	1 (5.0)
Food vendor	1 (5.0)
Laborer	1 (5.0)
Household size	
Mean (SD)	4.55 (2.64)
Range	1–10
Monthly income (currency)	
Mean (SD)	1440.00 (1266.66)
Range	0–4500
Primary income source, *n* (%)	
Business	12 (57.14)
Remittances	5 (23.81)
other	4 (19.05)
Farming	3 (14.29)
Salary/wages	2 (9.52)
Housing type, *n* (%)	
Compound house	14 (70.0)
Flat/apartment	4 (20.0)
Self‐contained	2 (10.0)
Main water source, *n* (%)	
Sachet/bottled	16 (80.0)
Pipe‐borne	2 (10.0)
Borehole	1 (5.0)
Other^b^	1 (5.0)
Sanitation facility, *n* (%)	
Flush toilet	9 (45.0)
Pit latrine	4 (20.0)
VIP latrine	6 (30.0)
Other^c^	1 (5.0)

Abbreviation: SD = standard deviation.

^a^“Higher” education was specified by participants and is distinct from “Tertiary” in this dataset.

^b^One participant’s response for water source was blank in the provided data.

^c^One participant’s response for sanitation facility was blank in the provided data. Percentages may not sum to 100 due to rounding.

### 3.2. Indigenous Knowledge and Food‐Based Coping Mechanisms for ART Side Effects

This analysis details the emergent themes on how participants utilize indigenous knowledge and specific food‐based strategies to manage the side effects of ART. The findings reveal that food is not merely consumed as a caloric requirement but is strategically employed as an active, therapeutic agent to buffer the body and mitigate adverse effects, thereby serving as a critical facilitator of adherence (Table 2).

**TABLE 2:** Themes and illustrative data on indigenous knowledge and food‐based coping mechanisms for ART side effects.

Theme	Subtheme	Identified codes	Participant excerpts (from parent data)
1. Proactive and Reactive Management of Side Effects	1.1. Using Specific Foods to Soothe Physical Discomfort	‐ Ginger for nausea	13002: *“I’ve also learned some kitchen staples like ginger tea can help.”*
		‐ Fats and proteins to buffer stomach	13004: *“If the pills upset my stomach, I’ll add a small piece of avocado or some groundnuts. It seems to soothe any discomfort.”*
		‐ Simple carbs for quick relief	12001: *“I used to feel nauseous and skip, but I learned that even a simple carb like cassava dough or gari helps with the side effects.”*
		‐ Sweet drinks for energy/dizziness	32014: *“It happens but rarely, but in situations like that I drink tea or Lucozade.”*
	1.2. Strategic Meal Timing and Composition	‐ Eating before medication as a non‐negotiable rule	13003: *“I sat on the bench in our yard, staring at the pills in my hand, thinking, “If I take these now, I’ll be sick on an empty stomach…” I ended up waiting until… my wife finally cooked a small pot of yam and sauce.”*
		‐ Prioritizing “heavy” or “small” foods based on sensation	13008: *“I always take my medications after eating. It helps me avoid feeling weak or dizzy. My routine is steady now, and I make sure to eat something before I take the pills.”*
		‐ Using available staples to create a protective meal	13006: *“I started feeling dizzy whenever I took it… so I went to prepare spaghetti to eat.”*

2. Indigenous Knowledge as an Adherence Strategy	2.1. Pragmatic Substitutions Using Local Pantry Staples	‐ Adapting meals based on availability and cost	12001: *“If we’re out of milk or bread, I switch to cassava dough or gari with water. I used to feel nauseous and skip, but I learned that even a simple carb helps.”*
		‐ Using traditional staples (gari, cassava, yam, beans) as a reliable base	32014: *“I usually cut down on food first. Maybe instead of cooking meat, we eat beans or garden eggs.”*
		‐ Knowledge of which local foods are “gentle” on the stomach	14001: *“My aunt sent me yams when I was out of stock, having that porridge staple kept me on schedule.”*
	2.2. Integrating Food‐Based Coping into Daily Routines	‐ Food strategies become habitual	12001: *“My pill tin sits next to my porridge pot. When I pour breakfast, I see the tin, instant reminder.”*
		‐ Knowledge is passed informally (self‐taught, peer‐shared)	32002: *“When I’m feeling anxious about money, I pray and sip bitter-leaf tea. It calms me enough to stick to my schedule.”*
		‐ Linking medication time with a specific food event	

3. The Intersection of Food Coping, Poverty, and Agency	3.1. Food Insecurity Forces Pragmatic, Not Ideal, Choices	‐ “Something is better than nothing” approach	32002: “*On those days I cut down on other things… and make sure the child eats. I’ll eat a little later or share what we have.”*
		‐ Coping mechanisms are born from scarcity	13003: *The decision to delay a dose until a “small pot of yam and sauce” was ready highlights that the quality and quantity of food matter, not just its presence*
		‐ Prioritizing children’s food, compromising own adherence buffer	
	3.2. Indigenous Knowledge as a Form of Patient Empowerment	‐ Participants are active agents in their care, not passive recipients	*The very act of discovering that ginger tea helps, or that avocado soothes the stomach, is an exercise of agency*
		‐ They experiment and discover what works for their bodies	*It is a low-cost, accessible solution developed within the community and household context, empowering individuals to manage their treatment within their means*
		‐ This knowledge provides a sense of control amidst structural constraints	

#### 3.2.1. Proactive and Reactive Management of Side Effects

Participants actively used specific local foods to prevent or alleviate the physical discomfort caused by ART. This was a common practice, with individuals identifying certain kitchen staples and common food items as effective remedies. For instance, ginger was explicitly used to combat nausea, while fatty or protein‐rich foods like avocado and groundnuts were consumed to create a protective buffer in the stomach before taking medication:

*“I′ve also learned some kitchen staples like* ginger tea *can help.” [13002, 45 years, Female]*

*If the pills upset my stomach, I’ll add a small piece of* avocado or some groundnuts. *It seems to soothe any discomfort.” [13004, 38 years, Female]*

Furthermore, the timing and composition of meals were strategically aligned with medication schedules. Taking ART on an empty stomach was widely avoided due to the fear of dizziness and weakness. Participants would often delay their dose until a meal, however small, was available, demonstrating that the *quality* and *presence* of food were as important as the medication itself for ensuring its ingestion:

*“I sat on the bench in our yard, staring at the pills in my hand, thinking, ‘If I take these now, I′ll be sick on an* empty stomach…’ *I ended up waiting until*… *my wife finally cooked a small pot of* yam and sauce.” *[13003, 52 years, Male]*

*“I always take my medications* after eating. *It helps me avoid feeling weak and dizzy.” [13008, 41 years, Female]*

#### 3.2.2. Indigenous Knowledge as an Adherence Strategy

The coping mechanisms employed were deeply pragmatic and rooted in the use of locally available and affordable pantry staples. When preferred foods were unavailable, participants seamlessly substituted them with traditional staples like gari (cassava flour), cassava dough, yams, and beans. This knowledge of which local foods are “gentle” on the stomach and can serve as a reliable base for medication was a key component of their adherence strategy:

*“If we’re out of milk or bread, I switch to* cassava dough or gari with water. *I used to feel nauseous and skip, but I learned that even a simple carb helps.” [12001, 44 years, Male]*

*“My aunt sent me* yams *when I was out of stock, having that porridge staple kept me on schedule.” [14001, 33 years, Female]*

These food‐based strategies were effectively integrated into daily routines, making adherence a habitual practice. For some, this involved linking pill‐taking directly to a specific meal, such as breakfast, thereby creating a natural and consistent reminder:

*“My pill tin sits next to my* porridge pot. *When I pour breakfast, I see the tin, instant reminder.” [12001, 44 years, Male]*

#### 3.2.3. The Intersection of Food Coping, Poverty, and Agency

The use of indigenous food knowledge often existed at the intersection of necessity and empowerment. Food insecurity forced pragmatic, rather than ideal, choices. Participants frequently described a “something is better than nothing” approach, where the choice of food was constrained by financial scarcity. This was poignantly illustrated by those who prioritized feeding their children, thereby compromising their own ability to take ART with an adequate meal:

“On those days I cut down on other things… and make sure the child eats. I′ll eat a little later or share what we have.” *[32002, 39 years, Female]*

Despite these constraints, the development and application of this knowledge was a powerful exercise of agency. Participants were not passive recipients of care but active agents who experimented with and discovered what worked for their bodies. The act of learning that ginger tea helps with nausea or that avocado soothes the stomach provided a sense of control and self‐efficacy, empowering individuals to manage their treatment within their limited means. This indigenous knowledge thus represents a low‐cost, accessible, and culturally resonant solution that is generated and sustained within the community and household context.

#### 3.2.4. Sources and Valuation of Indigenous Knowledge

Participants acquired food‐based coping knowledge from multiple sources, which they valued differently based on the perceived credibility, accessibility, and personal relevance of each source.

##### 3.2.4.1. Family Networks

The most frequently cited source of indigenous knowledge was family, particularly older female relatives. Participants described learning from mothers, aunts, and grandmothers who possessed generational knowledge of local food properties. *“My aunt sent me yams when I was out of stock, having that porridge staple kept me on schedule” [14001, 33 years, Female]*. This source was valued for its trustworthiness and the emotional support that accompanied the knowledge transfer.

##### 3.2.4.2. Peer Networks

Participants also learned from fellow PLHIV, particularly through informal conversations at clinic appointments or community gatherings. *“I’ve also learned some kitchen staples like ginger tea can help” [13002, 45 years, Female]*. Peer‐shared knowledge was valued for its practical relevance; it came from individuals facing the same challenges and had been tested in real‐world conditions.

##### 3.2.4.3. Personal Experimentation

Many participants described a process of trial and error, discovering what worked for their bodies through personal experience: *“I used to feel nauseous and skip, but I learned that even a simple carb like cassava dough or gari helps with the side effects” [12001, 44 years, Male]*. This self‐derived knowledge was valued for its personalized nature and the sense of agency it conferred.

##### 3.2.4.4. Healthcare Providers

While less frequently cited as a primary source of food‐based knowledge, healthcare providers were valued for their biomedical expertise and ability to validate or caution against certain practices. Participants who received counseling that acknowledged and built upon their existing practices reported feeling more supported in their adherence efforts.

##### 3.2.4.5. Hierarchy of Value

Participants placed the highest value on knowledge from sources that combined trustworthiness with practical applicability. Family knowledge was trusted but sometimes perceived as traditional rather than scientifically validated; peer knowledge was valued for its lived‐experience credibility; and provider knowledge was valued for its scientific authority but sometimes perceived as less accessible or culturally resonant.

### 3.3. Impact of Food‐Based Coping on PLHIV Wellbeing

The indigenous food‐based strategies employed by participants had demonstrable impacts on their physical, psychological, and social wellbeing.

#### 3.3.1. Physical Impact

Participants reported that using specific foods like ginger tea, avocado, and groundnuts significantly reduced the severity of ART‐induced nausea, dizziness, and gastric discomfort. As one participant noted, *“If the pills upset my stomach, I’ll add a small piece of avocado or some groundnuts. It seems to soothe any discomfort” [13004, 38 years, Female]*. This physical relief enabled participants to maintain their medication schedules without the debilitating side effects that often lead to treatment interruption.

#### 3.3.2. Psychological Impact

The ability to manage side effects through accessible, culturally familiar means provided participants with a sense of control and self‐efficacy: *“I used to feel nauseous and skip, but I learned that even a simple carb like cassava dough or gari helps with the side effects” [12001, 44 years, Male]*. This knowledge empowered participants, reducing anxiety around medication‐taking and fostering confidence in their ability to adhere to treatment.

#### 3.3.3. Social Impact

The sharing of food‐based knowledge within families and peer networks strengthened social bonds and created a supportive environment for adherence. Participants learned from family members (*“My aunt sent me yams when I was out of stock” [14001, 33 years, Female]*) and integrated these strategies into household routines, normalizing adherence within the domestic sphere.

## 4. Discussion

This study provides a detailed account of how individuals living with HIV in Ghana utilize indigenous knowledge and food‐based strategies to manage ART side effects, a crucial yet under‐documented facet of treatment adherence. The findings reveal a complex interplay between cultural wisdom, pragmatic coping, and patient empowerment within a context of socioeconomic constraint. The discussion below interprets these findings in relation to the wider body of literature.

### 4.1. Food as Active Pharmacological Buffer: Beyond Caloric Intake

The finding that participants use specific foods like ginger tea and avocado as active agents to manage ART side effects rather than merely as caloric intake is strongly supported by existing literature on food insecurity and HIV. This practice represents a critical form of “microsupport” that patients develop independently. Research consistently demonstrates that food insecurity is associated with decreased ART adherence, and that patients often develop strategic coping mechanisms to manage the challenging side effects of their medication [18]. The deliberate delay of a dose until food is available, a behavior observed in our study, directly reflects the difficult trade‐offs that food‐insecure individuals face, where the immediate physical harm of taking medication on an empty stomach outweighs concerns about slight dosing delays [18]. The physiological mechanisms underlying these food‐based strategies are increasingly supported by biomedical evidence. A randomized clinical trial conducted in Iran demonstrated that ginger supplementation significantly reduced ART‐induced nausea and vomiting, with only 56.4% of participants in the ginger group experiencing nausea compared to 90.2% in the placebo group (*p* = 0.001) [19]. This finding validates the indigenous practice of using ginger tea, reported by our participants, as an evidence‐based intervention for managing one of the most debilitating side effects of ART. Similarly, the use of fatty foods like avocado and groundnuts to create a “protective buffer” aligns with pharmacological principles: dietary fats can slow gastric emptying and reduce the peak concentration of certain drugs, thereby mitigating gastrointestinal irritation [20].

The pharmacological mechanisms underlying these food‐based strategies are multifaceted. Dietary fats, such as those found in avocado and groundnuts, can slow gastric emptying and reduce the peak plasma concentration of certain antiretroviral drugs, thereby mitigating gastrointestinal irritation and nausea [20]. This occurs because the presence of fat in the stomach delays the release of gastric contents into the small intestine, where most drug absorption occurs, resulting in a more gradual and less intense drug exposure that is better tolerated by the gastrointestinal tract. Additionally, ginger contains bioactive compounds such as gingerol and shogaol, which act as 5‐HT3 receptor antagonists and have anti‐inflammatory properties that reduce nausea and vomiting [19]. These compounds modulate the serotonin pathway, which is central to the emetic response, thereby directly counteracting the nausea induced by ART. The clinical significance of these mechanisms is substantial: when PLHIV can manage side effects through these accessible, culturally familiar means, they are more likely to maintain consistent adherence, achieve viral suppression, and prevent disease progression. Reduced side effects also improve quality of life, enabling individuals to engage in daily activities, maintain employment, and care for their families, thereby breaking the cycle of poverty and poor health [18]. These physiological effects translate directly into improved adherence, as participants who can manage side effects are more likely to maintain consistent dosing.

This finding is further corroborated by a study from Tunisia, which found that food insecurity was almost double among those who missed ART compared to those who adhered, indicating the fundamental role of food as a pharmacological buffer [21]. However, contrasting evidence suggests that not all patients develop these effective microsupport strategies independently. A study validating the Brief COPE instrument in China found that PLWH employed varying coping strategies, with some relying on maladaptive approaches that could compromise adherence [22]. This contrast may be explained by differences in cultural knowledge transmission or varying levels of social support networks that enable the development of these food‐based pharmacological strategies.

### 4.2. The Legitimacy and Efficacy of Indigenous Knowledge Systems

The use of local pantry staples like gari, cassava dough, and yams as effective coping mechanisms highlights how culturally embedded “microsupport” systems operate through pragmatic, culturally resonant knowledge. This aligns with a systematic review of food and nutrition interventions for Indigenous populations, which found that the most effective approaches combined educational strategies with food environment changes, recognizing the value of culturally appropriate solutions [23]. The impact of these indigenous strategies extends beyond symptom management to broader health outcomes. Food insecurity has been identified as a dominant barrier to ART adherence across sub‐Saharan Africa, with qualitative studies from the Democratic Republic of Congo revealing that patients explicitly linked hunger to nonadherence, stating, *“If I have nothing to eat, I get angry and push the pills bottle away from me”* [24]. By providing low‐cost, accessible solutions to the food‐adherence nexus, indigenous knowledge directly addresses this barrier. Furthermore, the integration of these practices into daily routines, such as placing the pill tin next to the porridge pot, creates behavioral cues that support consistent adherence, a strategy that aligns with behavioral science principles of habit formation [2]. The physiological and behavioral benefits of these strategies collectively support PLHIV’s ability to maintain virologic suppression and prevent disease progression.

Similarly, research on food literacy among Indigenous youth in Nepal emphasized that traditional food knowledge is a critical component of community food security and represents a sophisticated understanding of local food systems [25]. The efficacy of simple carbohydrates like gari in settling nausea finds support in nutritional science, which recognizes the gastric‐soothing properties of these staples [20]. Contrasting evidence, however, comes from studies of technology‐based adherence support approaches, where providers primarily favored text‐message reminders over more invasive monitoring systems [26]. This contrast suggests that while high‐tech “macrosupport” solutions are being developed in high‐income contexts, they may overlook the sophisticated low‐tech “microsupport” systems that already exist within cultural traditions. The difference may stem from a fundamental disconnect between biomedical approaches and indigenous knowledge systems, with the former often privileging Western scientific validation over culturally embedded practical knowledge.

The valuation of different knowledge sources observed in our study, with family networks being the most trusted, peers valued for lived experience, and providers respected for scientific authority, highlights the complexity of indigenous knowledge transmission. This finding aligns with research from Uganda, where traditional healers were found to be trusted community partners in HIV care because of longitudinal relationships and embedded cultural trust [27]. The preference for family and peer sources over formal healthcare providers for food‐based knowledge suggests that interventions aiming to leverage indigenous knowledge should work through existing community networks rather than attempting to replace them. Peer support interventions, which have been shown to enhance psychosocial support and improve treatment adherence among PLHIV [28], represent a promising vehicle for formalizing the sharing of food‐based coping strategies while respecting the organic, community‐driven nature of knowledge transmission.

The term “indigenous knowledge systems” in this context refers to the comprehensive, intergenerational body of knowledge, practices, and beliefs regarding the use of local food resources for health management. This system is not a single, monolithic entity but rather a dynamic and evolving set of practices that are transmitted through family networks, community elders, and peer interactions. It encompasses not only the identification of specific foods and their properties but also knowledge of preparation methods, optimal timing of consumption, and the cultural and spiritual significance of food. The impact of this system on PLHIV is direct and multifaceted. At the physical level, indigenous food knowledge provides PLHIV with accessible, low‐cost strategies to manage ART side effects, enabling them to maintain consistent adherence and achieve better clinical outcomes. At the psychological level, the ability to draw upon culturally familiar knowledge provides a sense of agency, control, and self‐efficacy, reducing anxiety and depression associated with chronic illness. At the social level, the sharing of this knowledge within families and communities strengthens social bonds and creates a supportive environment for adherence. Collectively, these impacts contribute to improved quality of life, treatment success, and long‐term health outcomes for PLHIV [6, 7].

### 4.3. Patient Agency and Empowerment in the Face of Structural Constraints

The demonstration of significant patient agency in our study, where participants actively experiment with and deploy culturally available resources to manage treatment challenges, presents a compelling narrative of resilience that challenges deficit‐based perspectives on adherence. This finding aligns with research on conditional economic incentives, which acknowledges that individuals make complex health decisions within constrained environments and often develop sophisticated strategies to navigate these challenges [29]. Similarly, a study in Mozambique that engaged traditional healers as adherence partners demonstrated how building on existing community resources, rather than imposing external solutions, led to high patient acceptance rates (94%), illustrating the power of culturally grounded agency [30]. Furthermore, research on cultural influences on ART adherence among Latino populations found that higher levels of bicultural self‐efficacy, the confidence to navigate different cultural contexts, were associated with better adherence, emphasizing the importance of patient empowerment in treatment success [31]. Contrasting evidence, however, comes from studies of food insecurity, which highlight how socioeconomic constraints can severely limit patient agency [18]. For instance, research with HIV‐infected injection drug users in British Columbia found that food insecurity was associated with frequent hunger and an inability to afford food, creating environmental constraints that overpower individual agency [32]. Additionally, a study in South Africa found that unconditional cash transfers had no direct effect on HIV outcomes, suggesting that individual agency alone may be insufficient without proper structural support [33]. These contrasts highlight the complex interplay between individual agency and structural constraints in shaping adherence behaviors.

### 4.4. The Double‐Edged Sword: Indigenous Knowledge Born From Scarcity

Our finding that indigenous coping strategies are frequently suboptimal adaptations to food insecurity rather than preferred choices highlights the paradoxical nature of these “microsupport” systems. This aligns with broader literature on food insecurity and HIV, which demonstrates a vicious cycle where food insecurity increases ART nonadherence, leading to worse health outcomes and deepened poverty [18, 34]. The painful prioritization of children’s food over one’s own medication buffer observed in our study is reflected in quantitative research from Uganda, where HIV‐affected households coped with food insecurity by reducing household meal portion sizes and selling nonproductive household assets [18]. Similarly, a recent assessment in Tunisia found that one‐third of PLHIV resorted to negative coping strategies such as begging and illegal activities to meet food needs, demonstrating how scarcity drives problematic adaptations [21]. Contrasting evidence comes from studies of more formal “macrosupport” interventions. A systematic review of food policies for Indigenous populations found that both targeted and universal policy actions could be effective, suggesting that the suboptimal microsupport strategies developed out of necessity could be complemented by structural interventions [23]. Additionally, a trial in Zimbabwe showed that modest economic incentives significantly increased HIV testing uptake, indicating that resource constraints, rather than cultural preference, may be driving the reliance on insufficient indigenous coping mechanisms [35]. These contrasts suggest that while indigenous knowledge provides crucial stopgap solutions, it is ultimately an inadequate substitute for comprehensive structural support.

### 4.5. Integrating Indigenous and Biomedical Systems for Holistic Care

The potential for synergistic relationship between indigenous food‐based practices and biomedical regimens observed in our study suggests a promising path toward more holistic HIV care. This aligns with successful adaptation efforts in Mozambique, where integrating traditional healers into the formal healthcare system through systematic translation of evidence‐based interventions resulted in high‐community acceptance and novel strategies to support ART adherence [30]. Similarly, a systematic review of food policies for Indigenous populations found that the most effective interventions combined educational strategies with policies targeting food price, composition, and availability, essentially integrating grassroot knowledge with structural support [23]. Furthermore, research on conditional economic incentives demonstrates how combining behavioral economics with traditional incentives can lead to improved HIV outcomes, showing the power of integrating different knowledge systems [29]. Contrasting evidence, however, comes from studies of technology‐based adherence support, where providers expressed concerns about approaches that might compromise patient autonomy or privacy, potentially damaging the patient–provider relationship [26]. Similarly, research on cultural influences on ART adherence found that bicultural self‐efficacy, not just integration of systems, was crucial for optimal outcomes, suggesting that mere structural integration is insufficient without attention to individual patients’ cultural navigation skills [36]. These contrasts highlight that successful integration requires more than simply adding traditional practices to biomedical frameworks; it necessitates a fundamental rethinking of how different knowledge systems can collaboratively support patient well‐being without compromising either cultural identity or clinical efficacy.

### 4.6. Implications

The findings of this study carry significant implications across research, policy, and clinical practice, advocating for a paradigm shift in HIV care. Theoretically, they reframe ART adherence as a socioecological practice and reposition patients from being “noncompliant” to “active agents” who leverage cultural tools, thereby contributing to the decolonization of health knowledge by validating indigenous foodways. Clinically, this necessitates moving beyond the generic instruction to “take with food” toward incorporating specific, nonjudgmental assessments of patients’ food‐based coping strategies and food insecurity into adherence counseling, validating effective indigenous practices to build trust and develop more realistic care plans. Programmatically and politically, this underscores the need to integrate indigenous knowledge into public health interventions, such as designing nutrition support with local ingredients and structuring peer groups for knowledge sharing, while also providing strong evidence for directly embedding food security interventions like livelihood programs and vouchers into HIV care, addressing the economic precarity that fundamentally underpins the reliance on these coping mechanisms.

## 5. Conclusion

This study has illuminated the critical role that indigenous knowledge and food‐based coping mechanisms play in enabling ART adherence among a population facing significant socioeconomic challenges. Participants demonstrated remarkable resilience and agency, strategically using local foods like ginger, avocado, and gari not just as sustenance, but as active agents to manage drug side effects. While these strategies are empowering and culturally resonant, they are often necessitated by and enacted within a context of poverty and food insecurity. The findings argue compellingly for a paradigm shift in HIV care, from a top‐down, purely biomedical model to a collaborative, patient‐centered approach that recognizes, respects, and integrates the valuable indigenous knowledge held by individuals and communities. Ultimately, supporting ART adherence requires not only providing the pills but also fostering the conditions, and recognizing the existing wisdom, that allows people to take them successfully.

## 6. Recommendations

Based on the study’s findings, we propose several key recommendations: for clinical practice, to develop and integrate a brief “Food and Side‐Effect Management” module into standard ART adherence counseling that proactively explores and validates patients’ existing coping strategies; for healthcare training, to equip nurses, counselors, and pharmacists with the skills to sensitively discuss indigenous knowledge and food insecurity; for peer support programs, to explicitly create space for sharing and documenting successful food‐based coping mechanisms to foster a community‐based repository of knowledge; and for future research, to conduct mixed‐methods or clinical studies that quantitatively evaluate the efficacy of specific indigenous strategies, such as ginger tea for nausea, to build a stronger evidence base for their formal integration into treatment guidelines.

## Author Contributions

Conceptualization: Kasim Abdulai and Ivan Addae‐Mensah. Methodology: Kasim Abdulai and Ivan Addae‐Mensah. Investigation: Kasim Abdulai, Angela Kyerewaa Aidoo, Mansura Ama Abakah, Adele Naa Ashikwei, and Mary Foriwaa Fosu. Formal Analysis: Kasim Abdulai, Ivan Addae‐Mensah, and Adjoa Antwiwaa Kye‐Duodu. Data Curation: Kasim Abdulai and Angela Kyerewaa Aidoo. Writing–original draft: Ivan Addae‐Mensah, Kasim Abdulai, and Angela Kyerewaa Aidoo. Writing–review and editing: Ivan Addae‐Mensah, and Kasim Abdulai. Supervision: Ivan Addae‐Mensah and Kasim Abdulai. Project administration: Ivan Addae‐Mensah and Kasim Abdulai.

## Funding

No funding was received for this manuscript.

## Disclosure

All authors have read and agreed to the published version of the manuscript. The affiliation of the corresponding author with HopeSpring Harvest Foundation is declared for transparency; however, the Foundation had no role in the design of the study; in the collection, analyses, or interpretation of data; in the writing of the manuscript; or in the decision to publish the results. The research was conducted and the manuscript prepared entirely independently, and the views expressed are solely those of the authors. This statement is made in accordance with the journal’s publication ethics policy.

## Conflicts of Interest

The authors declare no conflicts of interest.

## Data Availability

The data that support the findings of this study are available on request from the corresponding author. The data are not publicly available due to privacy or ethical restrictions.

## References

[1] Dirlikov E. , Kamoga J. , Talisuna S. A. et al., Scale-Up of HIV Antiretroviral Therapy and Estimation of Averted Infections and HIV-Related Deaths—Uganda, 2004–2022, MMWR. Morbidity and Mortality Weekly Report. (2023) 72, no. 4, 90–94, 10.15585/mmwr.mm7204a2. PMC9925129 36701255

[2] Zullig L. L. , Jazowski S. A. , Wang T. Y. et al., Novel Application of Approaches to Predicting Medication Adherence Using Medical Claims Data, Health Services Research. (2019) 54, no. 6, 1255–1262, 10.1111/1475-6773.13200. PMC6863234 31429471

[3] Magura J. , Nhari S. R. , and Nzimakwe T. I. , Barriers to ART Adherence in Sub-Saharan Africa: A Scoping Review Toward Achieving UNAIDS 95-95-95 Targets, Frontiers in Public Health. (2025) 13, 10.3389/fpubh.2025.1609743. PMC12185539 40556913

[4] Wohl D. A. , Kuwahara R. K. , Javadi K. et al., Financial Barriers and Lapses in Treatment and Care of HIV-Infected Adults in a Southern State in the United States, AIDS Patient Care and STDs. (2017) 31, no. 11, 463–469, 10.1089/apc.2017.0125. PMC5665096 29039984

[5] McAllister J. , Beardsworth G. , Lavie E. , MacRae K. , and Carr A. , Financial Stress is Associated With Reduced Treatment Adherence in HIV-Infected Adults in a Resource-Rich Setting, HIV Medicine. (2013) 14, no. 2, 120–124, 10.1111/j.1468-1293.2012.01034.x. 22780330

[6] Nachega J. B. , Adetokunboh O. , Uthman O. A. et al., Community-Based Interventions to Improve and Sustain Antiretroviral Therapy Adherence, Retention in HIV Care and Clinical Outcomes in Low- and Middle-Income Countries for Achieving the UNAIDS 90-90-90 Targets, Current HIV. (2016) 13, no. 5, 241–255, 10.1007/s11904-016-0325-9. PMC5357578 27475643

[7] Rhodes S. D. , Tanner A. E. , Mann-Jackson L. et al., Community-Engaged Research as an Approach to Expedite Advances in HIV Prevention, Care, and Treatment: A Call to Action, AIDS Education and Prevention: Official Publication of the International Society for AIDS Education. (2018) 30, no. 3, 243–253, 10.1521/aeap.2018.30.3.243. PMC6055521 29969308

[8] Kwame A. , Integrating Traditional Medicine and Healing Into the Ghanaian Mainstream Health System: Voices From Within, Qualitative Health Research. (2021) 31, no. 10, 1847–1860, 10.1177/10497323211008849. PMC8446885 33980093

[9] Peltzer K. , Preez N. F. , Ramlagan S. , and Fomundam H. , Use of Traditional Complementary and Alternative Medicine for HIV Patients in KwaZulu-Natal, South Africa, BMC Public Health. (2008) 8, no. 1, 10.1186/1471-2458-8-255. PMC2503977 18652666

[10] Laar A. K. , Lartey M. Y. , Ankomah A. et al., Food Elimination, Food Substitution, and Nutrient Supplementation Among ARV-Exposed HIV-Positive Persons in Southern Ghana, Journal of Health, Population and Nutrition. (2018) 37, no. 1, 10.1186/s41043-018-0157-x. PMC6278010 30509309

[11] Downer S. , Berkowitz S. A. , Harlan T. S. , Olstad D. L. , and Mozaffarian D. , Food is Medicine: Actions to Integrate Food and Nutrition Into Healthcare, 2020, 10.1136/bmj.m2482. PMC7322667 32601089

[12] Rezazadeh L. , Ostadrahimi A. , Tutunchi H. , Naemi Kermanshahi M. , and Pourmoradian S. , Nutrition Interventions to Address Nutritional Problems in HIV-Positive Patients: Translating Knowledge into Practice, Journal of Health, Population and Nutrition. (2023) 42, no. 1, 10.1186/s41043-023-00440-z. PMC10492288 37684634

[13] Peltzer K. , Friend-du Preez N. , Ramlagan S. , Fomundam H. , and Anderson J. , Traditional Complementary and Alternative Medicine and Antiretroviral Treatment Adherence Among HIV Patients in Kwazulu-Natal, South Africa, African Journal of Traditional, Complementary and Alternative Medicines. (2009) 7, no. 2, 125–137, https://pmc.ncbi.nlm.nih.gov/articles/PMC3021154/. PMC3021154 21304624

[14] Kiyimba T. , Yiga P. , Bamuwamye M. et al., Harnessing Traditional Knowledge and Experiences on Food Consumption in Uganda: A Mixed-Methods Study Exploring Indigenous Fruits and Vegetables With Potential Cardiometabolic Benefits, BMJ Public Health. (2025) 3, no. 2, 10.1136/bmjph-2024-001932. PMC12706100 41409765

[15] Prah J. , Hayfron-Benjamin A. , Abdulai M. , Lasim O. , Nartey Y. , and Obiri-Yeboah D. , Factors Affecting Adherence to Antiretroviral Therapy Among HIV/AIDS Patients in Cape Coast Metropolis, 2018, http://ir.ucc.edu.gh/jspui/handle/123456789/8172.

[16] Tarkang E. E. , Manu E. , Aku F. Y. et al., Healthcare Providers’ Experiences and Recommendations for Antiretroviral Therapy Adherence in Ghana: A Facility-Based Phenomenological Study, PLoS One. (2025) 20, no. 10, 10.1371/journal.pone.0333695. PMC12494291 41042774

[17] Eze R. A. , Sulaiman N. , Daud Z. ’A. M. , and Babadoko A. , Socio-Demographic and Food Insecurity Associated With Adherence to Antiretroviral Therapy Among HIV Adults in Ahmadu Bello University Teaching Hospital Zaria, Kaduna State Nigeria, African Health Sciences. (2023) 23, no. 4, 236–246, 10.4314/ahs.v23i4.26. PMC11225438 38974269

[18] Anema A. , Vogenthaler N. , Frongillo E. A. , Kadiyala S. , and Weiser S. D. , Food Insecurity and HIV/AIDS: Current Knowledge, Gaps, and Research Priorities, Current HIV. (2009) 6, no. 4, 224–231, 10.1007/s11904-009-0030-z. PMC5917641 19849966

[19] Dabaghzadeh F. , Khalili H. , Dashti-Khavidaki S. , Abbasian L. , and Moeinifard A. , Ginger for Prevention of Antiretroviral-Induced Nausea and Vomiting: A Randomized Clinical Trial, Expert Opinion on Drug Safety. (2014) 13, no. 7, 859–866, 10.1517/14740338.2014.914170. 24820858

[20] Wang Z. , Xu W. , Liu D. et al., Impact of Food Physical Properties on Oral Drug Absorption: A Comprehensive Review, Drug Design, Development and Therapy. (2025) 19, 267–280, 10.2147/DDDT.S497515. PMC11745047 39834644

[21] World Food Programme , Food Security and Nutritional Evaluation of People Living With HIV (PLHIV) in Tunisia, 2021, https://www.wfp.org/publications/2021-food-security-and-nutritional-evaluation-people-living-hiv-plhiv-tunisia.

[22] Cheng C. , Wang Q. , and Bai J. , Factor Structure of the Brief Coping Orientation to Problems Experienced Inventory (Brief-COPE) in Chinese Nursing Students, Nursing Reports. (2025) 15, no. 2, 10.3390/nursrep15020046. PMC11857923 39997782

[23] Amson A. , Zhang J. , Frehlich L. et al., Nutritional Interventions for Indigenous Adults in Canada-Opportunities to Sustain Health and Cultural Practices: A Scoping Review, International Journal of Circumpolar Health. (2024) 83, no. 1, 10.1080/22423982.2024.2418152. PMC11500539 39441950

[24] Musumari P. M. , Feldman M. D. , Techasrivichien T. , Wouters E. , Ono-Kihara M. , and Kihara M. , If I Have Nothing to Eat, I Get Angry and Push the Pills Bottle Away From Me: A Qualitative Study of Patient Determinants of Adherence to Antiretroviral Therapy in the Democratic Republic of Congo, AIDS Care. (2013) 25, no. 10, 1271–1277, 10.1080/09540121.2013.764391. 23383757

[25] Upreti Y. R. , Sharma T. , and Sharma P. , Indigenous Food and Nutrition Knowledge and Practice Among Ethnocultural Groups in Nepal: A Narrative Review, Mangal Research Journal. (2025) 6, no. 1, 63–78, 10.3126/mrj.v6i1.88189.

[26] Ogbechie M.-D. , Fischer Walker C. , Lee M.-T. et al., Predicting Treatment Interruption Among People Living With HIV in Nigeria: Machine Learning Approach, JMIR AI. (2023) 2, 10.2196/44432. PMC11041440 38875546

[27] Gogineni S. , Nuwagaba G. , Hooda M. et al., Traditional Healers Can Help Facilitate HIV Serostatus Disclosure: Results From a Qualitative Study in Rural Uganda, Journal of the International AIDS Society. (2025) 28, no. 10, 10.1002/jia2.70031. PMC12463694 40999574

[28] Kiirya Y. , Kitaka S. , Kalyango J. et al., Acceptability of an Online Peer Support Group as a Strategy to Improve Antiretroviral Therapy Adherence Among Young People in Kampala District, Uganda: Qualitative Findings, BMC Infectious Diseases. (2025) 25, no. 1, 10.1186/s12879-025-10831-8. PMC11969772 40181265

[29] Fahey C. A. , Njau P. F. , Dow W. H. , Kapologwe N. , and Mccoy S. I. , Effects of Short-Term Cash and Food Incentives on Food Insecurity and Nutrition Among HIV-Infected Adults in Tanzania: A Randomized Trial, AIDS. (2019) 33, no. 3, 515–524, 10.1097/QAD.0000000000002056. PMC6511258 30325776

[30] Audet C. M. , Pettapiece-Phillips M. , Tian Y. , Shepherd B. E. , Vermund S. H. , and Salato J. , If It Weren’t for My Traditional Healer, I Would Be Dead: Engaging Traditional Healers to Support People Living With HIV in Rural Mozambique, 2022, 10.1371/journal.pone.0270565. PMC9239464 35763519

[31] Robbins R. N. , D’Aquila E. , Morgello S. , Byrd D. , Remien R. H. , and Mindt M. R. , Cultural Influences on Antiretroviral Therapy Adherence Among HIV-Infected Puerto Ricans, Journal of the International Association of Physicians in AIDS Care. (2012) 11, no. 5, 314–322, 10.1177/1545109712453939. PMC3406236 22525858

[32] Anema A. , Weiser S. , Fernandes K. et al., High Prevalence of Food Insecurity Among HIV-Infected Individuals Receiving HAART in a Resource-Rich Setting, AIDS Care. (2011) 23, no. 12, 1604–1611, 10.1080/09540121.2011.582483. 21259135

[33] Pettifor A. , MacPhail C. , Hughes J. et al., The Effect of a Conditional Cash Transfer on HIV Incidence in Young Women in Rural South Africa (HPTN 068): A Phase 3, Randomised Controlled Trial, Lancet Global Health. (2016) 4, no. 12, 978–988, 10.1016/S2214-109X(16)30253-4. PMC5626439 27815148

[34] Tsai A. C. , Bangsberg D. R. , Emenyonu N. , Senkungu J. K. , Martin J. N. , and Weiser S. D. , The Social Context of Food Insecurity Among Persons Living With HIV/AIDS in Rural Uganda, Social Science & Medicine. (2011) 73, no. 12, 1717–1724, 10.1016/j.socscimed.2011.09.026. PMC3221802 22019367

[35] Kranzer K. , Simms V. , Bandason T. et al., Economic Incentives for HIV Testing by Adolescents in Zimbabwe: A Randomised Controlled Trial, The Lancet HIV. (2018) 5, no. 2, 79–86, 10.1016/S2352-3018(17)30199-3. PMC5809636 29170030

[36] Quinn K. G. and Voisin D. R. , ART Adherence Among Men Who Have Sex With Men Living With HIV: Key Challenges and Opportunities, Current HIV. (2020) 17, no. 4, 290–300, 10.1007/s11904-020-00510-5. PMC7363570 32557117

